# Swimming Using a Unidirectionally Rotating, Single Stopping Flagellum in the Alpha Proteobacterium *Rhodobacter sphaeroides*

**DOI:** 10.3389/fmicb.2022.893524

**Published:** 2022-06-01

**Authors:** Judith P. Armitage

**Affiliations:** Department of Biochemistry, University of Oxford, Oxford, United Kingdom

**Keywords:** *Rhodobacter*, motor, swimming, chemotaxis, model

## Abstract

*Rhodobacter sphaeroides* has 2 flagellar operons, one, Fla2, encoding a polar tuft that is not expressed under laboratory conditions and a second, Fla1, encoding a single randomly positioned flagellum. This single flagellum, unlike the flagella of other species studied, only rotates in a counterclockwise direction. Long periods of smooth swimming are punctuated by short stops, caused by the binding of one of 3 competing CheY homologs to the motor. During a stop, the motor is locked, not freely rotating, and the flagellar filament changes conformation to a short wavelength, large amplitude structure, reforming into a driving helix when the motor restarts. The cell has been reoriented during the brief stop and the next period of smooth swimming is a new direction.

## Introduction

One of the distinguishing features used to identify bacterial species in the days before genomics was the number and position of their flagella and pattern of swimming. When we first started investigating the behavior of the small coccobacillus-shaped purple non-sulfur alphaproteobacterium *Rhodobacter sphaeroides* (*R. sphaeroides*), it was clear that it only had one flagellum, but unlike the better studied monoflagellate species, it did not change direction by briefly reversing its swimming direction, but rather it occasionally just stopped swimming. The stopped cell is buffeted about by the local environment, so that when the cell starts to swim again it is usually pointing in a new direction.

It had been shown that bacterial motility is driven by the electrochemical ion gradient across the bacterial membrane, with either the proton (pmf) or the sodium motive force driving rotation of the flagellar motor depending on the species (Manson et al., 1977). Howard Berg had also shown that under high external load, the speed of the motor is linearly related to the size of the pmf, while at low loads, the motor reaches maximum speed at low pmf values (Meister and Berg, 1987). The pmf in most bacterial species tends to be maintained at a high level, even under adverse metabolic conditions to ensure bacterial survival. These experiments suggest that under adverse conditions, motility is one of the last physiological activities to stop, indeed probably running even after the ATP synthase has stopped or reversed activity to keep a residual pmf across the membrane. The *R. sphaeroides* flagellar motor is driven by the pmf. In early experiments, we reduced the pmf of photosynthetically growing cells using ionophores and found the bacteria stopped swimming in the dark, but regained motility when illuminated by actinic light. Intriguingly, it could take minutes for the cells to start swimming after the light was switched back on, leading us to speculate that the motor structure might disassemble when the pmf driving force was removed, something we only discovered to be true 20 years later (Armitage et al., 1985). The loss of the pmf led to a loss of swimming, so we needed to be sure that the motor really stops when the pmf is high. Two experiments confirmed that the motor really stops while maintaining a pmf, while another experiment suggested that loss of the pmf might alter the motor stability.

**FIGURE 1:**
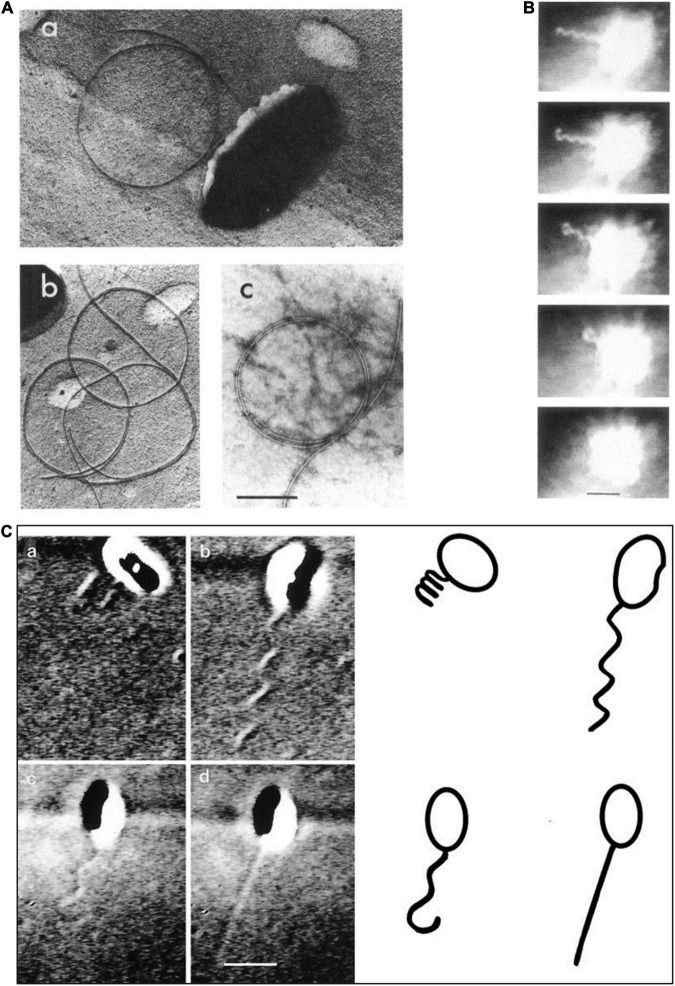
Images of the Rhodobacter *sphaeroides* (*R. sphaeroides*) flagellum. **(A)** Various electron micrographs showing relaxed non-functioning filament. **(B)** Still images from dark-field light microscopy of swimming cell showing filament coiling against cell body after a stop. **(C)** Differential interference contrast (DIC) light microscopy images of swimming cells showing different conformations (a) coiled, (b) swimming, (c) starting to relax during a stop, and (d) fast rotating probably small amplitude filament. From Armitage and Macnab (1987) and Armitage et al. (1999) with permission.

Back in the early 1980s, it was not possible to see a bacterial flagellum on a living, swimming bacterium because the diameter of the filament is below the resolution of a standard light microscope. However, Bob Macnab at Yale, building on some early experiments carried out by Pijper in South Africa (Pijper, 1955), had built a dark-field light microscope that uses extremely bright light. Light scattering allowed the filament to be seen on living, swimming cells, although only at a distance from the cell body as scattering of very high-intensity light made the cell body appears as a superbright ball. Using a modified version of this microscope, we saw the *R. sphaeroides* single flagellar filament rotate and push the bacterium forward and occasionally abruptly stop (Armitage and Macnab, 1987). When the bacterium stopped the functional helical filament collapsed from the distal end of the filament, coiling into a short wavelength, large amplitude form against the cell body (Figure 1B). After a brief stopped period, the smaller amplitude, longer wavelength conformation of the filament reformed from the cell body outward to the filament tip when the cell started to swim again, usually in a new direction. This suggested that motor rotation is needed to force the flagellar filament flagellin subunits into a conformation that forms a waveform that allows swimming. Early study examining the structure of the flagellin subunits in a filament suggests that they can form 12 filament waveforms dependent on the forces on the filament, with only a limited number able to push a cell through it environment. The experiments visualizing the *R. sphaeroides* flagellum suggested a waveform allowing swimming only occurs when the motor rotates (Kamiya et al., 1982). Interestingly, in both the negative-stained electron micrographs and in the high light dark-field experiments, there were large numbers of free circular structures, which we now realize were detached filaments collapsed into the relaxed waveform (Figure 1A). Again, this suggests that only the torque force caused by motor rotation can alter the interaction of the flagellin monomers to drive formation of a functional waveform.

The second experiment confirming the *R. sphaeroides* stop-start motor used antiflagellin antibody to tether cells to a glass slide by their flagella (Poole et al., 1988). Experiments with *Escherichia coli* (*E. coli*) had used such experiments to confirm that bacteria use a rotary motor to drive flagellar rotation (Silverman and Simon, 1974). Tethering *E. coli* by short filaments or hooks resulted in cell bodies rotating smoothly in one direction and occasionally switching the direction of rotation, reflecting the smooth swimming that occurs with counterclockwise rotation and a direction changing tumble that occurs with a brief period of clockwise rotation. The *R. sphaeroides* single motor, however, only rotates in one direction, counterclockwise, stopping occasionally, and then resuming counterclockwise rotation. Occasional cells have been seen to rotate clockwise, but no cell has ever been seen to switch its direction of rotation (Packer and Armitage, 1993).

## Genomics and Structure

**FIGURE 2:**
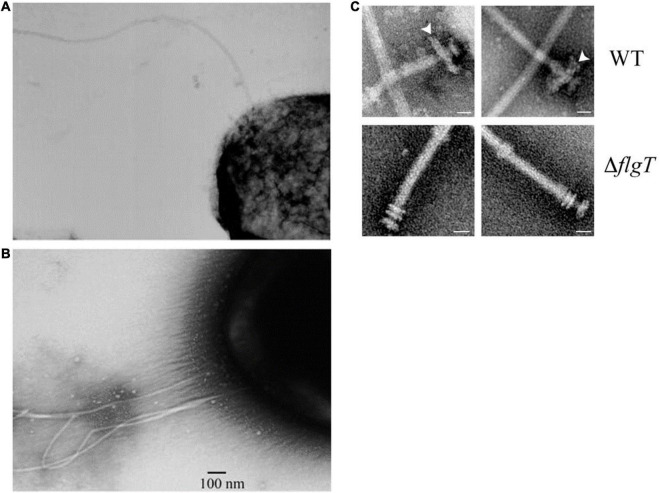
Electron micrographs showing **(A)** single unidirectional flagellum expressed under laboratory conditions, Fla1. **(B)** Polar tuft of flagella, Fla2 expressed from CtrA controlled regulon when Fla1 is repressed. **(C)** Additional ring associated with L/P ring of Fla1, lost in FlgT mutants. Taken from Poggio et al. (2007) and Fabela et al. (2013) with permission.

It now seems that *R. sphaeroides* flagella have a complex evolutionary history. In the laboratory, *R. sphaeroides* has only been seen to swim using a single, unidirectionally rotating flagellum; genome sequencing has revealed that it has 2 complete flagellar regulons, Fla1 and Fla2 (Mackenzie et al., 2001; Poggio et al., 2007; Porter et al., 2011b). The laboratory of George Dreyfus has been able to express the second set of flagella genes in mutants unable to express the Fla1 unidirectional flagellum (Figure 2A). These mutants express a polar tuft of filaments, similar to polar tufts seem on other alpha proteobacteria (Figure 2B). The expression of the Fla2 regulon is dependent on the Ctr regulatory system known to control lifestyle switching and motility in many alpha proteobacteria (de la Mora et al., 2015; Vega-Baray et al., 2015; Poncin et al., 2018). Genome sequencing also revealed that *R. sphaeroides* encodes 3 chemotaxis pathway operons. Two of these operons are expressed under laboratory conditions and regulate the activity of the Fla1 unidirectional motor (Porter et al., 2002). The third operon, resembling in organization of the chemosensory operons found in related alpha proteobacteria, is not normally expressed, but appears to be able to interact with the motor of the Fla2 polar tuft of flagella (Martinez-del Campo et al., 2011). These operons encoding the normally unexpressed flagella and chemotaxis system does not appear to be degenerate and are, therefore, probably expressed under specific environmental conditions; however, these have yet to be identified. It has been suggested that the Fla2 flagellar system and controlling CheOp1 are probably the ancestral system and Fla1 and *CheOp2* and *CheOp3* genes were acquired by horizontal gene transfer (Hernandez-Valle et al., 2020).

The Fla1 single, unidirectional flagellum driving bacterial swimming in the laboratory is encoded by a completely separate regulon whose expression is controlled by a sigma 54 transcription factor (Pena-Sanchez et al., 2009). The stopping frequency of the motor is controlled by two different chemosensory operons (Porter et al., 2008b). The genes of the different operons cannot complement mutations in the other flagella or chemosensory systems (Scott et al., 2010). Interestingly, most polar flagella are precisely positioned by sets of targeting proteins, e.g., TipN/TipF, HupP, or FhlF (Schuhmacher et al., 2015). There are no obviously polar targeting genes associated with the expressed Fla1 unidirectional filament. In our early observations, we described the filament as subpolar, as we never saw it at the poles. Later, painstaking analysis by Chi Aizawa of large number of electron micrographs suggested that these single flagella are in fact randomly positioned (personal communication), rather like the peritrichous filaments of *E. coli*, but with the regulon expressed only once per cell cycle. When following dividing cells, a new filament is only seen on the new daughter cell and it is very rare to see two filaments within a single body length. The new filament starts to rotate just before cells separation and results in doublets of cells spinning on their axes, until the cells separate and swim off in different directions.

Adding to the idea that the active *R. sphaeroides* flagellum evolved from a different bacterial group is the unusual structure of the hook. Images of most peritrichously flagellate bacteria show a curved hook linking the rod to the flagellar filament. The hook monomers ordered to interface through hook-associated proteins with the flagellin subunits and enabled the torque to be transmitted from the rotating motor to the filament, driving the helical conformation of the flagellum. Driving the interactions between the flagellin molecules in the 11 protofilaments that make up the hollow filament causes it to form a helix rather than straight tube and drives the switch between different wavelengths and handedness upon a switch in direction of motor rotation. The hook shape is thought to allow the 6 or more filaments to come together as a bundle to push the cell forward (Samatey et al., 2004; Brown et al., 2012). The periodic switch in motor rotational direction drives a change in hook waveform and, thus, filament waveform and this forces the bundle apart and causes the bacterium to tumble transiently. *R. sphaeroides* has a straight rather than bent hook and a diameter smaller rather than larger than the filament (Figure 2C). Although there does not seem to have been an extensive survey, the hook of polar flagellate species such as *Caulobacter crescentus* and *Campylobacter jejuni* also appears to be straight (Wagenknecht et al., 1981; Matsunami et al., 2016). This may suggest that the Fla1 flagellum thought to be acquired by horizontal gene transfer may have been polar, but because the associated polar positioning protein was lost, it is now positioned randomly. The unidirectional motor means that on a stop, the torque normally forcing the hook and then the flagellin subunits into interactions resulting in helical conformations is lost and the structure returns to a minimal energy state. Measuring rotation rates of tethered cells suggests that the motor can change periodically speed, something seen in some other related species such as *Sinorhizobium* (Poole and Armitage, 1988; Packer and Armitage, 1994; Attmannspacher et al., 2005). The reason for speed change is unclear, particularly as the driving pmf is expected to be stable. Using early differential interference contrast (DIC) microscopy to visualize the flagella of free swimming cells, we saw some cells transiently swimming very fast that appeared to be have short wavelength and small amplitude helical filaments. This suggests that *R. sphaeroides* has at least 3 patterns of movement, long periods of medium speed, smooth swimming punctuated by short periods of fast swimming and stops (Figure 1C; Armitage et al., 1999). How this complex pattern of behavior impacts movement in the natural environment is unclear.

Swimming *R. sphaeroides* cells have usually been studied in buffer and, therefore, with a low load on their motors; it is known that in the environment, they can form extensive biofilms. While electron micrographs of the isolated motors look similar to the basic motors of enteric species, the Dreyfus laboratory has identified additional motor proteins, an H-ring plus FliL and MotF. These appear to sit in the periplasmic region of the motor close to the outer membrane L/P ring, but unlike related FliL proteins, they appears to interact with the stator and be essential for motility (Figure 2C; Suaste-Olmos et al., 2010; Ramirez-Cabrera et al., 2012; Camarena and Dreyfus, 2020). The basic flagellar motors of species such as *E. coli* have a series of rings in the cytoplasmic and outer membrane. On the cytoplasmic face of the FliG rotor, ring sits on the FliM/FliN switch complex. FliG is associated with the MS ring, embedded in the cytoplasmic membrane. FliG rotation is driven by the ring of peptidoglycan anchored MotA/MotB stator proteins. These are the site of proton or sodium transport. Ion flow changes the structure of the Mot stators and, thus, the electrostatic interaction with the FliG ring and drives rotation. A rod protein is attached to the rotor and spans the periplasm, passing through the outer membrane L and P rings. A type 3 secretion system ATPase sits inside the FliM/FliN ring, a switch ring, and exports the unfolded proteins of the hook and then filament through the rod to polymerize at the distal end of the filament (Armitage and Berry, 2020). Cryoelectron tomography has revealed that while this is the basic motor architecture, species that need to produce a higher torque, for example, species that move through very viscous environments often have extra rings or rings with a larger diameter to produce increased torque (Beeby et al., 2016). The motors of species driven by sodium motive forces also have additional rings. Why *R. sphaeroides* has an extra ring is unclear. While it is clear that the *R. sphaeroides* Fla1 motor rotation is driven by the pmf, *R. sphaeroides* can be isolated from brackish water and is tolerant of high sodium concentrations as well as being able to form extensive biofilms. While loss of the pmf does cause swimming to stop, supporting the hypothesis that Fla1 is a proton driven motor; further investigation is required to understand the possible role of these additional motor rings in different environments.

## Analysis of Swimming Patterns Persistence and Active Direction Changing

**FIGURE 3:**
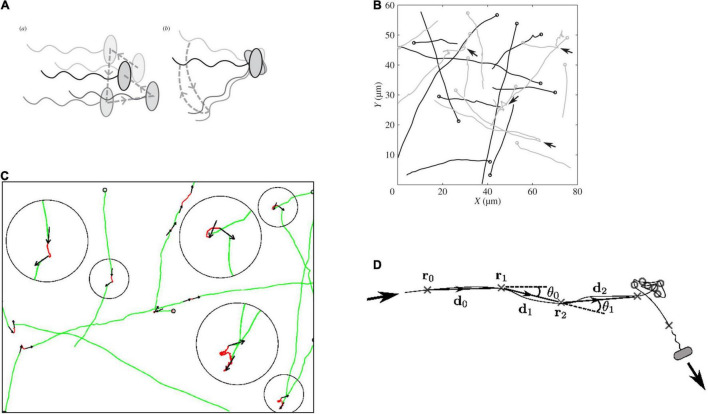
Measurement of *R. sphaeroides* free swimming tracks. **(A)** Difference between rotational Brownian diffusion during a stop (a) vs. active reorientation (b). **(B)** Tracks with arrows marking stops, **(C)** details of movement during stops, and **(D)** analysis of angles changed while free swimming and stops (details in Rosser et al., 2013, 2014).

**FIGURE 4:**
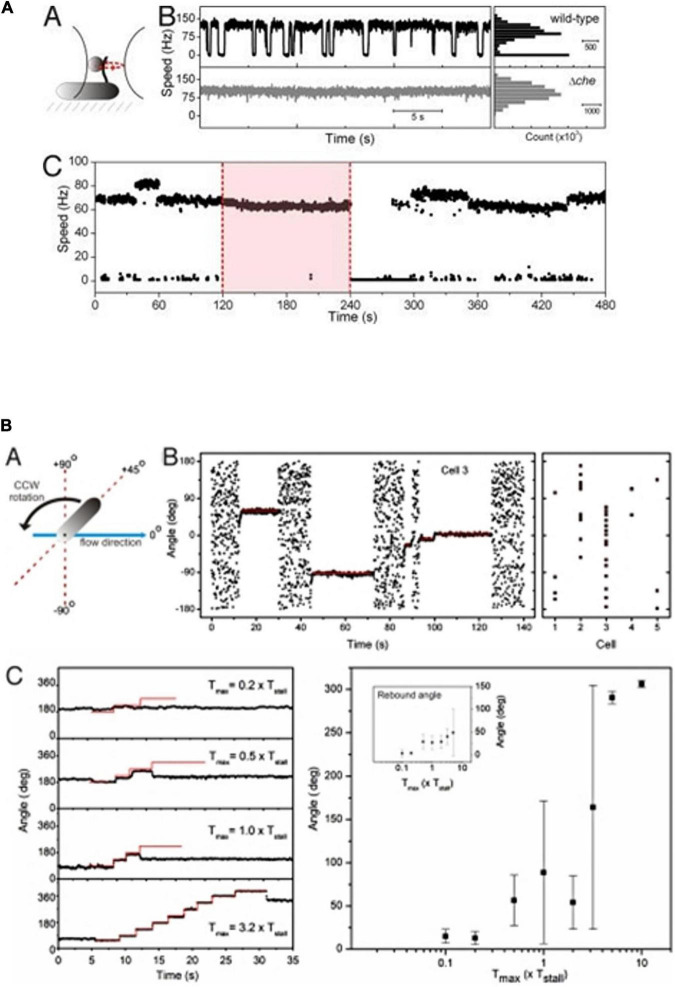
Tethered cell analysis of *R. sphaeroides* motor behavior. **(A)** Behavior of bead attached to a truncated filament of a wt and a non-chemotactic mutant. Wt motors show long periods of smooth swimming with occasional stops, while non-chemotactic mutants show constant smooth swimming. (C) Longer timescale shows a tethered wt cell rotating smoothly with occasional increases in speed. Pink box shows addition of attractant and loss of stops, removal results in stopped cell and adaptation. **(B)** Shows cell tethered by its flagellum and stopped by large chemosensory stimulus and the force required to rotate the cell (details in Pilizota et al., 2009).

Detailed analysis of the swimming patterns of bacterial populations in 3 dimensions, with each bacterium following a random swimming pattern was, and still is, challenging. Over the years, both the microscopy and tracking software able to follow multiple cells simultaneously have been steadily improved. The simultaneous tracking and analysis of trajectories in greater detail have provided increased understanding of *R. sphaeroides* swimming patterns (Figure 3). Tethering and free swimming data both show that the *R.* sphaeroides motor has an unstimulated motor bias of 0.85, i.e., it spends most of the time rotating, driving, swimming, with only very occasional stops. This is similar to the free swimming bias of *E.* coli swimming with a bundle of flagella where the cells swim for more time than they tumble; however, a single tethered *E.* coli motor has a bias closer to 0.5 making behavioral responses easier to measure (Berry and Armitage, 2000; Figure 4A). The high motor bias makes it hard to measure a response to a positive stimulus and much easier to identify a stimulus-related stop. Detailed analysis of swimming tracks suggests that while there is directional persistence during stops, there needs to be more than just Brownian rotational motion for reorientation, suggesting that either the hook might change angle or, more likely, as the motor starts to rotate at the end of a stop, the reforming functional waveform being pushed down the length of the filament by the increasing torque reorients the cell body (Figure 3; Rosser et al., 2013, 2014).

## Brake vs. Clutch

How does the motor stop and when it stops does it brake or declutch? Do the stators disengage from the rotor allowing free spinning without proton flow or do they lock to the rotor, possible allowing continued proton flow without rotor movement.

In *E. coli* and indeed most bacterial species, direction changing is caused by the binding of the phosphorylated chemosensory protein CheY to the FliM component of the flagellar motor switch (Wadhams and Armitage, 2004). As mentioned above, the structure of the motor resembles that of the simple *E. coli* motor, but with the addition of an extra periplasmic ring. Recent structural studies have revealed details of the complete structure of the *E.* coli and *Salmonella* flagellar motors and suggested mechanisms for switching mechanisms (Johnson et al., 2021; Tan et al., 2021). Unfortunately, there are no detailed molecular structures of the *R. sphaeroides* motor proteins to allow comparisons between a switching and stopping motor. Unlike the simple chemosensory system of *E. coli* with one signaling CheY protein, *R. sphaeroides* expresses 3 CheY proteins under normal laboratory conditions. Two of these CheYs, CheY3 and CheY4, are regulated by a large membrane spanning cluster of chemotaxis proteins, assumed to sense the extracellular concentration of chemoeffectors, while the 3rd protein, CheY6, is regulated by a large complex of chemosensory proteins in a single cytoplasmic cluster, localized to the surface of the nucleoid (Porter et al., 2011a; Jones and Armitage, 2015, 2017). The phosphorylation state of CheY6 is regulated by an unusual CheA kinase divided into 2 separate proteins, CheA4 acts as the kinase and when active phosphotransfers to a His residue on CheA3, which phosphotransfers only to CheY6 and an adaptation protein CheB2 (Porter et al., 2008a). CheA3 also has a dephosphorylation domain, exclusively removing phosphoryl residues from CheY6-P. The activity of CheA3 and CheA4 is regulated by two cytoplasmic receptors, assumed to sense the metabolic state of the cell and balance the kinase to dephosphorylation rate and, thus, control the level of CheY6-P in the cytoplasm (Beyer et al., 2019).

**FIGURE 5:**
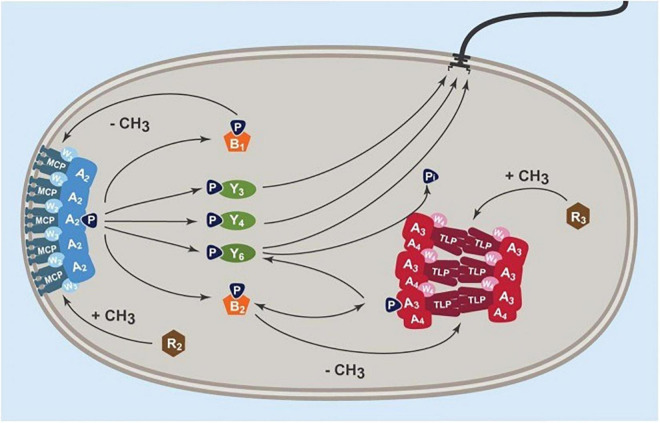
Current model of chemosensory control of *R. sphaeroides* Fla1 motor stopping. Signals from the transmembrane chemosensory cluster, MCP, W3, and A2 control the phosphorylation state of CheY3 and CheY4. These compete with CheY6 whose phosphorylation state is controlled by the kinase CheA4 and the phosphatase CheA3 through the cytoplasmic chemoreceptor TlpT. Each cluster has an independent adaptation system consisting of a constitutive methyltransferase (CheR) and a constitutive methylesterase (CheB) regulated by the associated histidine kinase, CheA (from Beyer et al., 2019).

CheY6-P is the only CheY able to stop the motor, although all the CheYs can bind the motor and CheY6 and either CheY3 or CheY4 is required for chemotaxis (Porter et al., 2006). Deletion of CheY6 results in smooth swimming, while deletion of CheY3 and CheY4 results in wild-type swimming patterns, but no chemotaxis. Models suggest that there is steady-state phosphorylation of the phosphotransfer site of CheA3 by CheA4, but signals through the cytoplasmic receptor TlpT regulate CheA3 and specifically the activity of the dephosphorylation domain, which dephosphorylated CheY6-P. TlpT is the only cytoplasmic chemoreceptor showing adaptation (Beyer et al., 2019). A steady state level of CheY6-P controls the unstimulated stopping frequency of the *R. sphaeroides* motor. A study by Dreyfus suggested that *in vitro*, both the phosphorylated and unphosphorylated CheYs can bind the motor (del Campo et al., 2007). As only CheY6-P stops the motor, it suggests that during a fall in chemoeffector concentration, there must be an increase in CheY6-P concentration causing motor stopping. It is likely that steady state levels of CheY3 and CheY4 compete with CheY6-P for binding to the motor allowing periods of swimming and stopping as concentrations fluctuate. A fall in a metabolic chemoeffector causes an increase in CheY6-P levels, probably by a reduction in dephosphorylation activity and it outcompetes the other CheYs for FliM, stopping the motor. Adaptation of TlpT, controlling CheA3 activity, then resets the dephosphorylation state and the level of CheY6-P returns to normal and CheY3 and CheY4 can again compete and allow periods of free swimming (current model shown in Figure 5; Beyer et al., 2019). Unfortunately, while there has been detailed structural analysis of FliM and changes that occur to CheY∼P binding in enteric bacterial species, we have no detailed knowledge of the structure of FliM in *R. sphaeroides* or how 3 CheY homologs can bind FliM, but only one stop motor rotation.

To identify whether a stop caused by CheY6-P binding locks the motor or released the stator proteins, *R. sphaeroides* was tethered by their flagella to microscope slides and the motors stopped using a large chemotactic stimulus. Using either viscous flow or attaching a bead to the flagellum and holding that bead in an optical trap, we measured the force needed to make a stopped motor rotate. The force needed to rotate the cells was compared to the stall torque of the motor under swimming conditions, i.e., the force needed to stop a smoothly rotating motor. Using either method, the force required was 2–3 times the stall torque of the motor, showing that the motor is locked (Figure 4B; Pilizota et al., 2009). This is supported by the fixed angles measured during stops, suggesting that there is no drift in the stopped motor. Interestingly, measurement of the stiffness of the tether, the hook, in these experiments, shows it is an order of magnitude stiffer than that of *E. coli*, consistent with the straight rather than curved hook.

As mentioned above, the FliG ring rotates against a ring of MotA/MotB stator proteins. However, we showed that in *E. coli*, the stators are in constant exchange with stators diffusing in the cytoplasmic membrane. Individual stators only staying associated for about 30 s and the number associated linked to the external force on the motor. Each stator engages individually, with the numbers engaged increases as the external viscosity increases. This suggests that the off-rate changes as the force on the motor changes, presumably transmitted through the filament, and hook from the external environment. When the *R. sphaeroides* motor locks during a stop, the stators were found to still be exchanging, suggesting that the release of stators from the peptidoglycan required to exchange is independent from their engagement to the rotor.

## Summary

While the basic structure of bacterial flagellar motors appears to be conserved through evolution, there are obvious variations on a theme. Species such as *R. sphaeroides* are extremely metabolically and structurally diverse, with some activities mutually exclusive. It can grow anaerobically, using either anaerobic respiration or photosynthesis; under these conditions, its cytoplasm is filled with vesicles and it can fix nitrogen, if complex nitrogen becomes limiting. It can also grow aerobically, with organic acids, as its favored carbon source. However, although components of the electron transport chain are shared between aerobic respiration and photosynthesis, the two systems are, as with most photosynthetic bacteria, mutually exclusive. Has this metabolic complexity led to the unusual swimming behavior seen in this species? It seems probably that the ancestral species swam using a tuft of polar filaments controlled by a classical chemosensory system, as these look similar to those of several other related species. However, during evolution, *R. sphaeroides* appears to have acquired not only a second flagellar system, but 2 chemosensory pathways. The chemosensory pathways now interplay to allow *R. sphaeroides* to make a “decision” about whether to respond to a specific metabolite by controlling the activity of very unusual single, stopping flagellar motor. We can only speculate about why this is the flagellum and control system we see expressed in the laboratory and why it is unable to switch direction of rotation. It works, *R. sphaeroides* swims rapidly through liquid environments, at speeds of over 50 μms^–1^, responding to changes in light, oxygen, and a wide range of organic acids by modulating the stopping frequency of the motor. Despite years of trying, we have not been able to get both the systems to express together. We need to work out when this Fla1 system is repressed and the second Fla2 system expressed to really understand how this bacterium exploits it environment to the full.

## Author Contributions

JA wrote the manuscript.

## Conflict of Interest

The author declares that the research was conducted in the absence of any commercial or financial relationships that could be construed as a potential conflict of interest.

## Publisher’s Note

All claims expressed in this article are solely those of the authors and do not necessarily represent those of their affiliated organizations, or those of the publisher, the editors and the reviewers. Any product that may be evaluated in this article, or claim that may be made by its manufacturer, is not guaranteed or endorsed by the publisher.
